# Phase transformation path in Aluminum under ramp compression; simulation and experimental study

**DOI:** 10.1038/s41598-022-23785-7

**Published:** 2022-11-08

**Authors:** Lijie He, Danae Polsin, Shuai Zhang, Gilbert W. Collins, Niaz Abdolrahim

**Affiliations:** 1 Materials Science Program, University of Rochester, Rochester, NY 14627 USA; 2 Departments of Mechanical Engineering, University of Rochester, Rochester, NY 14627 USA; 3 Laboratory for Laser Energetics, University of Rochester, Rochester, NY 14623 USA; 4 Department of Physics and Astronomy, University of Rochester, Rochester, NY 14627 USA

**Keywords:** Phase transitions and critical phenomena, Structural properties

## Abstract

We present a framework based on non-equilibrium molecular dynamics (NEMD) to reproduce the phase transformation event of Aluminum under ramp compression loading. The simulated stress-density response, virtual x-ray diffraction patterns, and structure analysis are compared against the previously observed experimental laser-driven ramp compression in-situ x-ray diffraction data. The NEMD simulations show the solid–solid phase transitions are consistent to experimental observations with a close-packed face-centered cubic (fcc) (111), hexagonal close-packed (hcp) structure (002), and body-centered cubic bcc (110) planes remaining parallel. The atomic-level analysis of NEMD simulations identifiy the exact phase transformation pathway happening via Bain transformation while the previous in situ x-ray diffraction data did not provide sufficient information for deducing the exact phase transformation path.

## Introduction

The advancement in experimental techniques has drastically improved our understanding of solid-phase stability and solid–solid phase transformation under high pressure. The development of gas gun^1^, pulsed-power^2^, and laser drivers^3^, combined with in situ x-ray diffraction (XRD)^4,5^, unveiled the structure and phase information of numerous materials under dynamic, high-pressure, shock, and quasi-isentropic compression with strain rates ranging from 10^4^ to 10^8^ s^−1^. In situ XRD is capable of capturing the Debye–Scherrer diffraction cones of the sample at different pressures and projecting these diffraction cones into $2\theta -\phi$ space, where the Bragg angle $\theta$ is the angle between the X-ray beam and the family of lattice planes and $\phi$ is the azimuthal angle around the incident x-ray direction. The $2\theta$ profile can be used to calculate the interplanar distance according to Bragg’s law^6^. The angle $\chi$, which is the angle between the sample norm and plane norm can be calculated using the equation^7^ $\mathrm{cos}\left(\chi \right)=\mathrm{cos}\left(\phi \right)/\mathrm{cos}(\theta )$ and used to evaluate the crystallographic texture during the phase transformation by tracking which planes remain parallel. This technique have been successfully applied to understand high-temperature/high-pressure physics such as twinning and lattice dynamics in laser-driven shocked tantatlum^8^, high-pressure phase stability during decompression in zinc ferrite nanoparticles^9^, and phase transformation pathway from graphite to hexagonal diamond^10^.

In a recent work by Polsin et al.^11^, in situ XRD were utilized to detect the crystal structure of Aluminum (Al) under ramp compression loading. The authors found out that a solid–solid phase transition, consistent with a transformation to a hexagonal close-packed (hcp) structure, occurs at around 216 GPa, while a transformation to a structure consistent with the body-centered cubic (bcc) structure occurs at 320 GPa. The results of in situ XRD suggested that the close-packed face-centered cubic (fcc) (111), hcp (002), and bcc (110) planes remain parallel through the solid–solid fcc–hcp and hcp–bcc transformations. However, the mechanism and pathway of the phase transformation upon dynamic compression, which has recently emerged as an important and interesting topic in high-pressure research^11–14^, remain unclear. Experimentally, this would require time-resolved diffraction measurements during the laser-driven shock/ramp compression, which is technically challenging. However, even in situ XRD crystallography is not sufficient to determine the exact phase transformation pathway from high-pressure high-temperature experiments, since multiple transformation paths can potentially produce the similar parallel planes during transformation^15^. With the aid of non-equilibrium molecular dynamics (NEMD), the exact atomistic configuration of the structure at each stage during the NEMD simulation of ramp loading can be determined at the atomistic level. The virtual XRD profiles can also be easily obtained and directly compared with experiments to verify the simulations. Thus, NEMD simulations will provide a fundamental understanding of the plastic deformation mechanisms and structural phase transformation pathway and XRD profiles will be used for experimental verification.

NEMD is a tool that is suitable for studying irreversible macroscopic processes, according to the second law of thermodynamics, such as spallation^16^, shock loading^17^, and ramp loading^18,19^. However, traditional NEMD suffers from the critical drawback of only applicable to systems with a limited temporal/spatial scale due to the high computational cost^20^. This drawback is even more prominent in ramp loading studies, where the experimental strain rate is slow (less than 10^6^ s^−1^). In order to achieve lower ramp rates in NEMD, longer simulation durations are required. In turn, larger material systems are also needed to allow stress wave development and propagation. These limitations put a heavy burden on the computational cost, justifying the need to reduce the system size without compromising the capability to predict the physics of deformation. Thus, dynamic scaling of the ramp loading system has been proposed and scrutinized^18,21^ to overcome this problem. Previous work by Thompson et al.^21^, as well as Lane et al.^18^, have demonstrated that NEMD in a reduced-size system is fully capable of capturing all of the elastic and most of the plastic response during ramp loading of a much larger system, providing that the time and position are scaled by the same factor while compression rate dx/dt is held constant. This theory enabled the computational investigation of ramp-compression experiments on the spatial and temporal dimensions of microns and nanoseconds to be conducted in a reduced system on the scale of nanometers and picoseconds, which is approachable by NEMD simulations. Details of this method and its applicability are discussed in Sect. 1 of the Supplementary Materials.

In this manuscript, we use NEMD simulations with dynamic scaling to study phase transformations of ramp compressed Al. The interactions between Al atoms are modeled using the embedded atom method (EAM) potential developed by Winey et al.^22^. This potential was specifically developed to investigate the high-pressure physics of Al and is widely used in shock-related simulations^16,23–25^. Specifically, Yang et al.^26^ have used Winey potential to reproduce the Hugoniot curves, Grüneisen coefficient, and melting temperature of Al under shock loading up to 300 GPa and found great agreements with experiments. We will then revisit our previous findings on the structural phase transformation mechanism of Al, where it undergoes fcc to bcc transitions under laser-driven ramp compression with in situ XRD^15,27^. It is found that the NEMD simulations can reproduce the stress-density response of the experiment exceptionally well when the simulation adopts the same scaling factor for both temporal and spatial dimensions compared to the experiment (e.g., 1/20 of the experiment). Then, based on the XRD pattern analysis, atomistic snapshots analysis, and local lattice orientation calculation, we discuss the structural phase transformation path featuring a dislocation-assisted Bain transformation. Finally, the virtual XRD of the structure at the different stages and the relative relationship between the close-packed planes are compared with the experimental observation, showing a good agreement in the phase transformation signatures.

## Simulation setup

For the NEMD setup, an initial < 001 > -oriented 10.12 nm × 10.12 nm × 1000 nm single crystal (SC) Al system with 6.25 × 10^6^ atoms and scaling factor of 1/20 of the experiment is created and ramp-compressed in the Z direction. Moving pistons are set initially at the lower Z boundary and move up with linearly increasing velocity up to 6 km/s in 500 ps. Periodic boundary conditions are applied along the transverse directions. A momentum mirror that reflects the momentum of any atom that comes in contact with it, is applied at the higher Z boundary. Other SC setups with different scaling factors (i.e., structure dimension and loading acceleration rate) are compared in detail in the Supplementary Materials. A texturized (< 001 > -oriented) nanocrystal (NC) structure is also generated via Voronoi tessellation^28^. In order to allow dislocations to pile up and interact with each other in the nanograins, the average grain size is set to be 15 nm, and subsequently, the NC structure dimension is set at 30.37 nm × 30.37 nm × 100 nm with a total atom count of 5.6 × 10^6^. Thus, the acceleration duration is set at 50 picoseconds (ps) to fulfill a scaling factor of 1/200 for both time and length. The plasticity contributor in texturized NC Al is discussed in “Plasticity contributor in texturized nanocrystalline Al” of this manuscript.

## Results and discussion

### Stress-density response and atomistic deformation path

**Figure 1:**
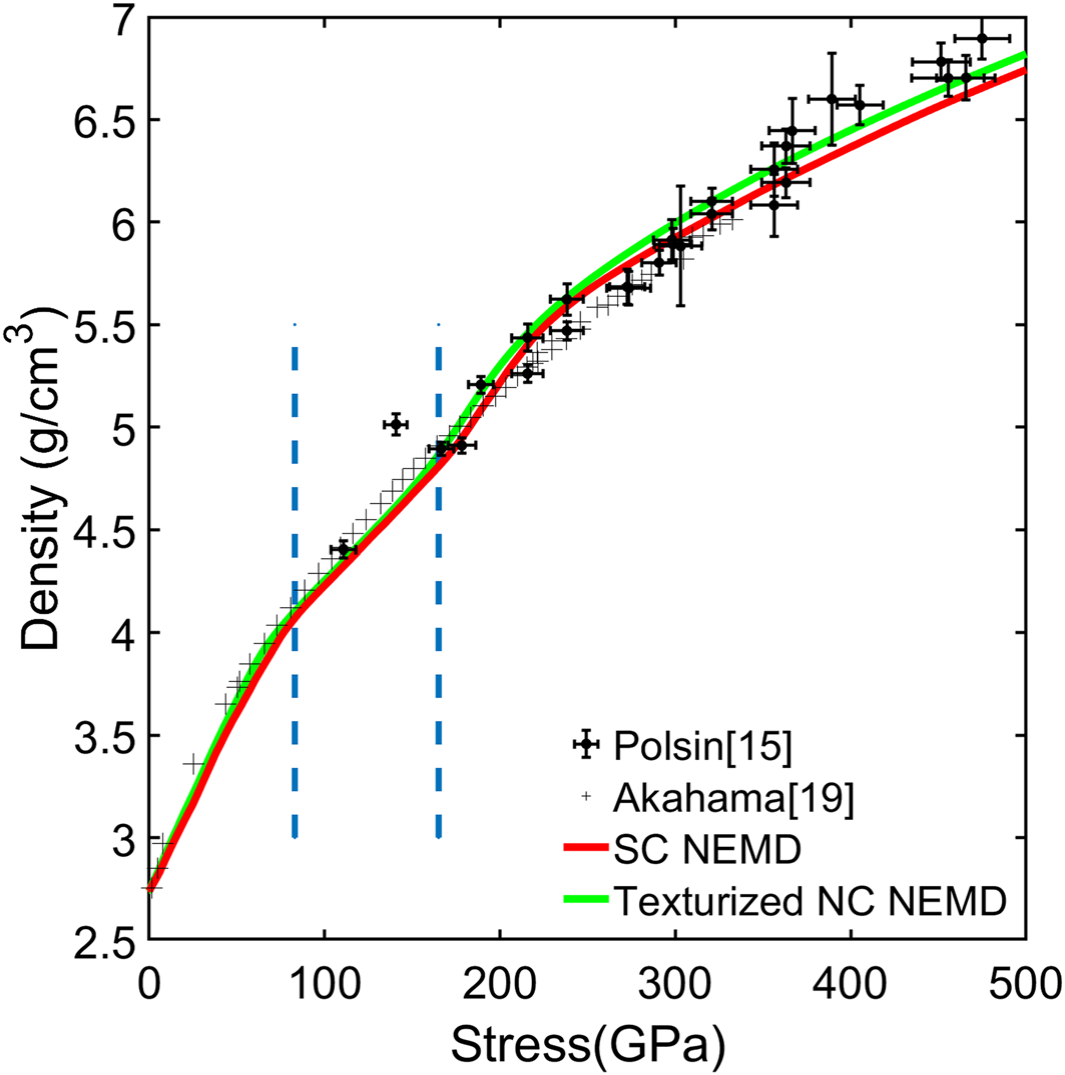
Density–stress curves for different simulation setups. The data are compared with ramp experiment from Ref.^15^ and diamond anvil cell data from Ref.^19^. For both the simulation data and experiment data, the stress and density refers to the average stress and density throughout the thickness of the entire Al domain as a function of time, which will be referred to as the global stress and density for the rest of the paper. The first vertical dashed bar denotes the pressure onset for 100% bcc in SC NEMD. In between the two vertical dashed bars, the structure is full bcc and deforming elastically for SC NEMD. After the second dashed bars, defect growth is observed in SC NEMD.

**Figure 2:**
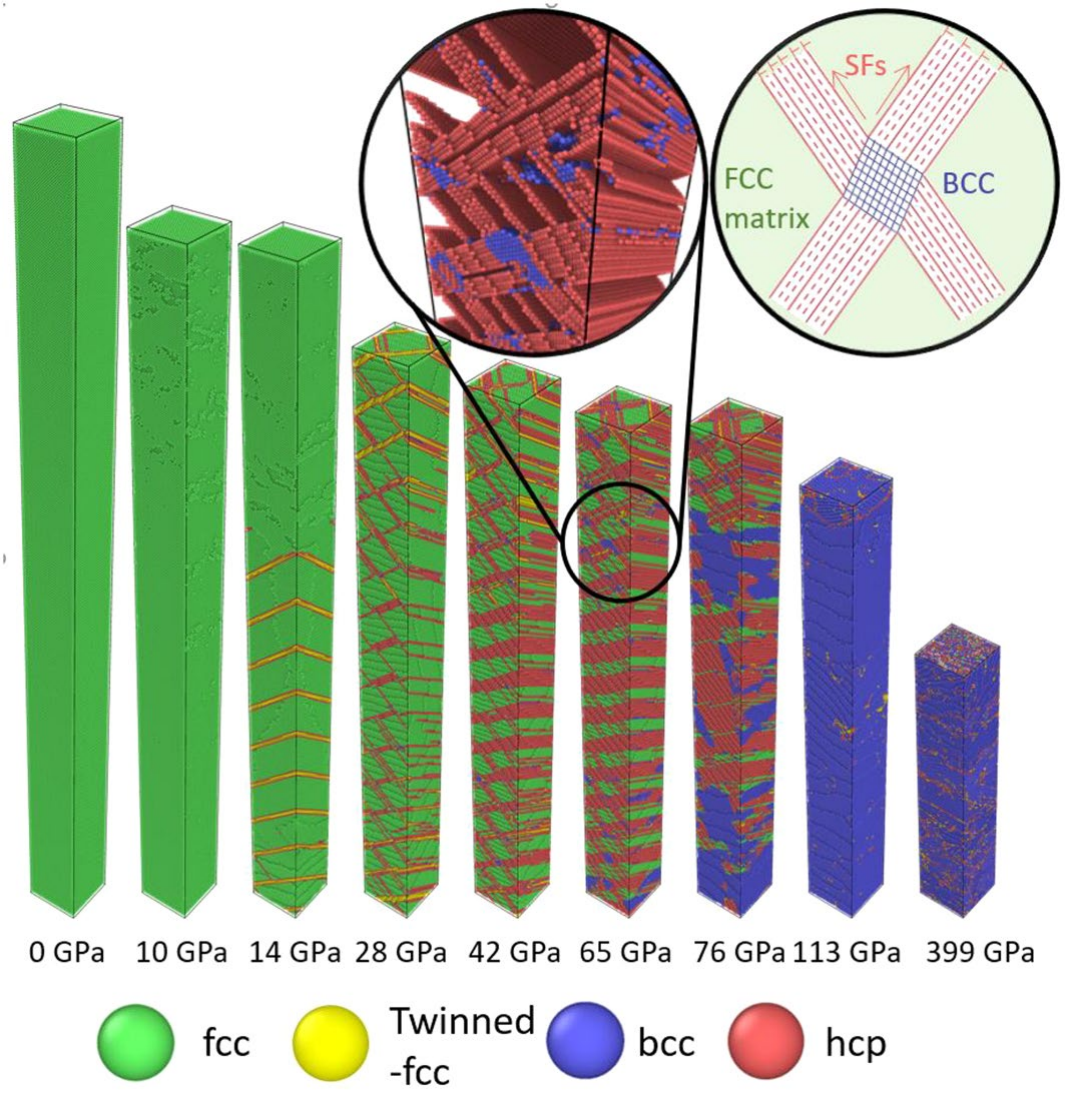
Atomistic snapshot of a representative slice from SC NEMD at different global stress states. The loading direction is from bottom to top. The magnification of the circled area shows the initial bcc nucleation, where fcc atoms are not displayed for clarity.

Figure 1 demonstrates the similarity of the stress-density response between the NEMD simulations, laser-assisted ramp loading compression, and diamond anvil cell data. The similarity only persists when the NEMD simulation adopts the same temporal and spatial scaling factor (i.e., 1/20 for SC structure and 1/200 for NC structure). A dimensionless strain rate ${\dot{\widetilde{v}}}_{p}$ is proposed by Lane et al.^18^, to identify systems that are correctly scaled:

$${\dot{\widetilde{v}}}_{p}=\frac{{v}_{t}L}{\tau {C}_{0}^{2}}$$

where ${v}_{t}=6 km/s$ is the terminal velocity for both the experiment and the simulation. $\tau$ denotes the acceleration duration, $L$ is the piston length and ${C}_{0}=6.27 km/s$ is the ambient sound velocity of Al. When a simulated system adopts the same temporal and spatial scaling factors as the experiment, its dimensionless strain rate will be the same as the experiment, and it serves as a good scaled representation of the experiment setup. When the dimensionless strain rate is different, the behavior of the system will start to deviate from the experiment. This is especially obvious when the dimensionless strain rate is larger than a certain threshold, i.e., temporal scaling factor being too large or spatial scaling factor being too small. Under this situation, the scaled setting will approach the shock regime, where the structure will exhibit different stress-density response compared to the shockless ramp loading experiment, shown in Fig. 1. The readers can refer to the Supplementary materials for a more detailed discussion on the fundamentals of the scaling method and NEMD simulation results under different dimensionless strain rates. In this paper, all data shown will be from systems with the same dimensionless strain rate as the experiment in reference^11^. Figure 2 shows a series of snapshots of a centerpiece from the SC structure under ramp loading NEMD simulation at different timesteps. The structure goes through the following stages. From 0 to 10 GPa, this centerpiece goes through elastic deformation. At 14 GPa, micro twin faults (i.e., thin twin faults with only 3–4 atomic layers) form along the $(111)$ slip plane. It should be noted that the micro twins only form in the SC system due to the specific orientation and small cross sectional area of the SC system. This mechanism is almost absent in the NC structure with grains in random orientations which is more consistent with real experimental structures, see details in “Plasticity contributor in texturized nanocrystalline Al”. At 28 GPa, new leading Shockley partials start to nucleate and propagate, leaving stacking faults (SFs) along the $(\overline{1 }11)$ planes behind. When these $(\overline{1 }11)$ SFs intersect with the $(111)$ micro twins; the micro twins get unzipped and transform into $(111)$ SFs. With further ramp compressing, the $(111)$ SFs thicken until around 65 GPa, when bcc phase starts to nucleate in certain parts of the structure. The bcc phase first nucleates at or in the vicinity of the intersecting SFs on $(\overline{1 }11)$ and $\left(111\right)$ slip planes, see the illustration in the magnified circle in Fig. 2. This mechanism for new bcc phase nucleation is similar to the Olson-Cohen model^29^ that describes the austenite to α’-martensite phase transformation facilitated by the SFs observed in iron-based alloy^30–32^. The bcc phase then proliferates, surpasses SFs thickening, and becomes the dominant mechanism at around 80 GPa. At around 90 GPa, the structure fully transforms into bcc. The nature of this phase transformation will be discussed in detail in “Plasticity contributor in texturized nanocrystalline Al”. After the phase transformation, the structure deformed elastically from 90 to 165 GPa, corresponding to a nearly linear response at a reduced slope in the stress-density curves (between the blue dotted line in Fig. 1). Then as defects start to nucleate in the bcc phase above 165 GPa, the stress-density curve changes slope again.

### Characterization of ramp-loaded Al microstructures using virtual X-ray diffraction

**Figure 3:**
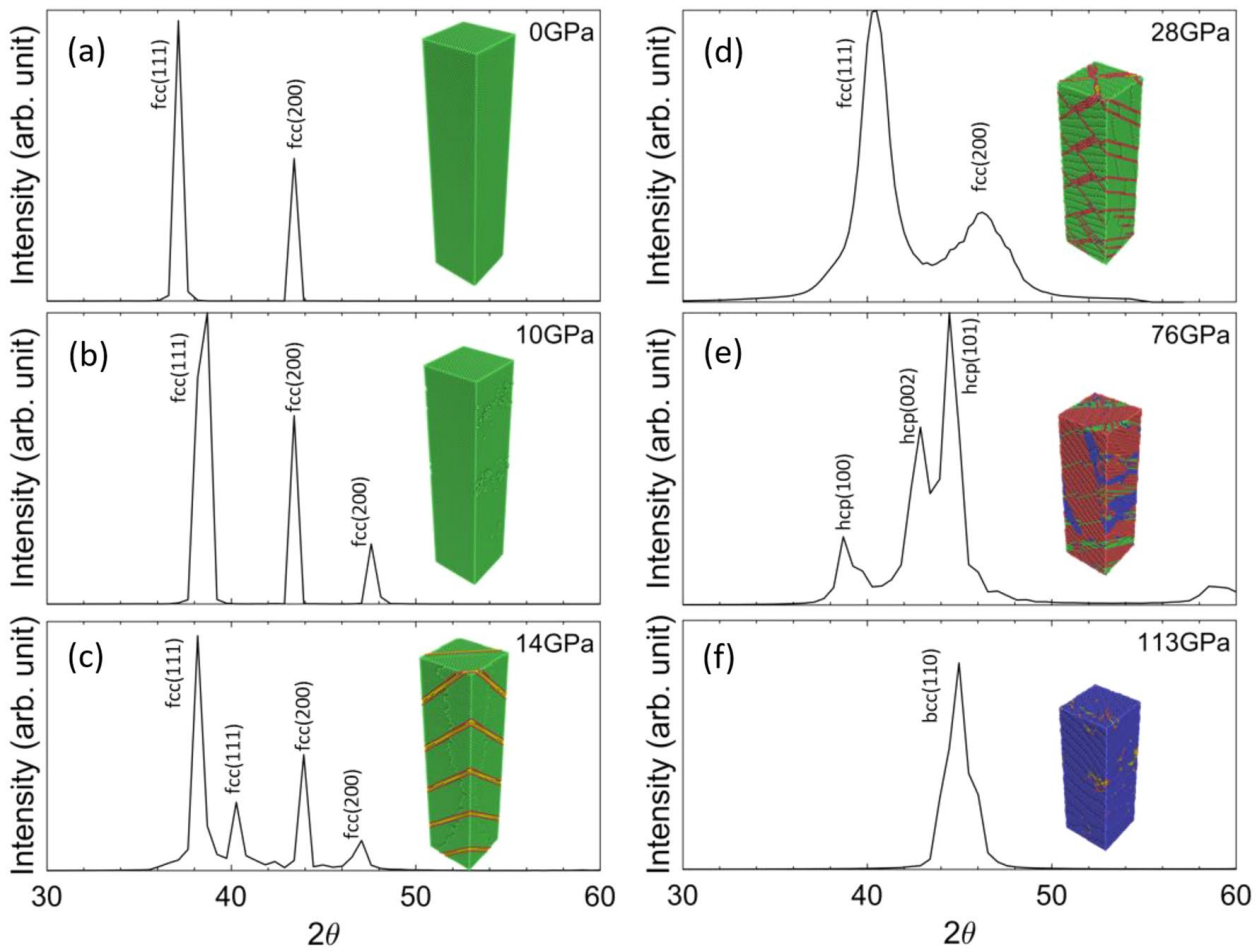
X-ray diffraction patterns for a representative slice (atomistic snapshots shown as insets and colored in the same manner as Fig. 2) from SC NEMD at different stress state.

The snapshots in Fig. 2, demonstrated the atomistic details of dislocation and twinning activity, and phase process. While such atomistic details can not be captured in real experiments, the virtual X-ray diffraction of simulations can be directly compared between simulation and experimental results. Thus, understanding the diffraction signature and establishing its corresponding relation to the atomistic picture will help in revealing the mechanisms of phase transformation from the in situ diffractogram acquired during experiments. The characterization of the virtual XRD for the single-crystal structure is considered in this section and the comparisons with real XRD data are discussed in “Phase transformation path and comparison between simulation and experiments”. We take the snapshots of a representative slab, which has a length of 50 nm at 0 GPa, as illustrated in the insets of Fig. 3, and characterize this region using virtual XRD as it undergoes different elastic/plastic stages under the ramp loading.

The snapshot at 0 ps represents the initial unstrained, equilibrated microstructure. As shown in Fig. 3a, the XRD pattern for this snapshot demonstrates sharp peaks. The diffraction angle ($2\theta$ angle) for the first and second peaks of the XRD profile occurs at 37.13° and 43.41°, matching the reported value of the {111} and {200} peaks of fcc Al, Fig. 3a. As the ramp loading starts, the uniaxial compression along the [001] direction will cause the $(002)$ and $(00\overline{2 })$ peaks shifting to larger angles, while the $\left(200\right),$ $\left(\overline{2 }00\right),$ $\left(020\right),$ $(0\overline{2 }0)$ peaks remain unchanged. This difference in straining of {200} planes results in the splitting of the {200} peak at 10 GPa, as shown in Fig. 3b. On the contrary, all {111} planes have the same angle with the loading direction and are thus compressed in the same magnitude, which results in the {111} peak shifting to a larger angle instead of splitting. Starting from 14 GPa, we begin to see micro twin formation in the region along with the $\left(111\right)$ planes, Fig. 3c. Interestingly, a splitting of the {111} peaks is also observed associated with the occurrences of the micro twins. The splitting is due to the micro twins not only accommodating a significant amount of atomic strain but also allowing the elastic strain in the fcc phase to be redistributed in a non-uniform manner. A closer inspection of the atomic picture reveals the $\left(111\right)$,$\left(\overline{1 }11\right),$ and $\left(\overline{1 }1\overline{1 }\right)$ planes remained unstrained. This corresponds to part of the first peak with no shifting from 10 to 14 GPa, Fig. 3c. On the contrary, the $\left(\overline{1 }\overline{1 }1\right)$ plane is now more compressed with a new inter-plane distance of 2.15 Å instead of 2.26 Å, which corresponds to the split of the {111} peak where its second part occurred at a higher diffraction angle (second peak in the XRD). It should be noted again that microtwins only form in the SC structure due to small cross sectional area of this system where limited space is available for accommodation of plastic deformation. The micro twins are almost absent in experiments and in NC structure. Nevertheles, this study shows that the XRD profiles can capture the twin formations via peak splitting. As stated in the previous section, starting at 17 GPa, new SFs along with the $(\overline{1 }11)$ slip planes also start to nucleate and unzip the $\left(111\right)$ micro twins into $\left(111\right)$ SFs when coming into contact. Then at 28 GPa, the split {111} peaks recombined into one single peak, suggesting all {111} peaks are compressed at the same magnitude, and homogeneous straining in the fcc matrix is achieved again, Fig. 3d. In the meanwhile, significant broadening of the $\left(111\right)$ and $\left(200\right)$ peak is observed. More interestingly, there exists a discrepancy between the lattice constant calculated from the (111) peak (3.70 Å) and (200) peak (3.77 Å), suggesting the two peaks have shifted relatively. Sharma et al.^33^ have investigated similar XRD profiles obtained during ramp compression of gold based on the theoretical work of Warren^34^ and concluded that the relative shifting of the peaks could only be due to the presence of SFs while broadening could be related to multiple mechanisms, including SFs, twinning, size broadening, and strain broadening. Thus, the existence of SFs in the structure can also be concluded from the 28 GPa XRD profile besides its corresponding atomistic configuration. As the strain goes on, the $\left(111\right)$ stacking faults continue to thicken in certain parts of the structure. The thickened SFs have an hcp configuration with its basal planes (001)_hcp_ parallel to (111)_fcc_ slip planes. The XRD profile for this specific snapshot also exhibits hcp signature as illustrated in Fig. 3e. It is also noticed that even at the peak of the SFs thickening event (at around 76 GPa), only 41.2% of the total atoms are part of the SFs. This agrees with the observation that SFs thickening is not uniform in the structure. Then within 15 GPa, phase transformation becomes dominant and surpasses the SFs thickening globally. At 113 GPa, the bcc phase propagates across the entire structure, leading to an XRD profile exhibiting the bcc Al signature, Fig. 3f.

### Phase transformation path and comparison between simulation and experiments

**Figure 4:**
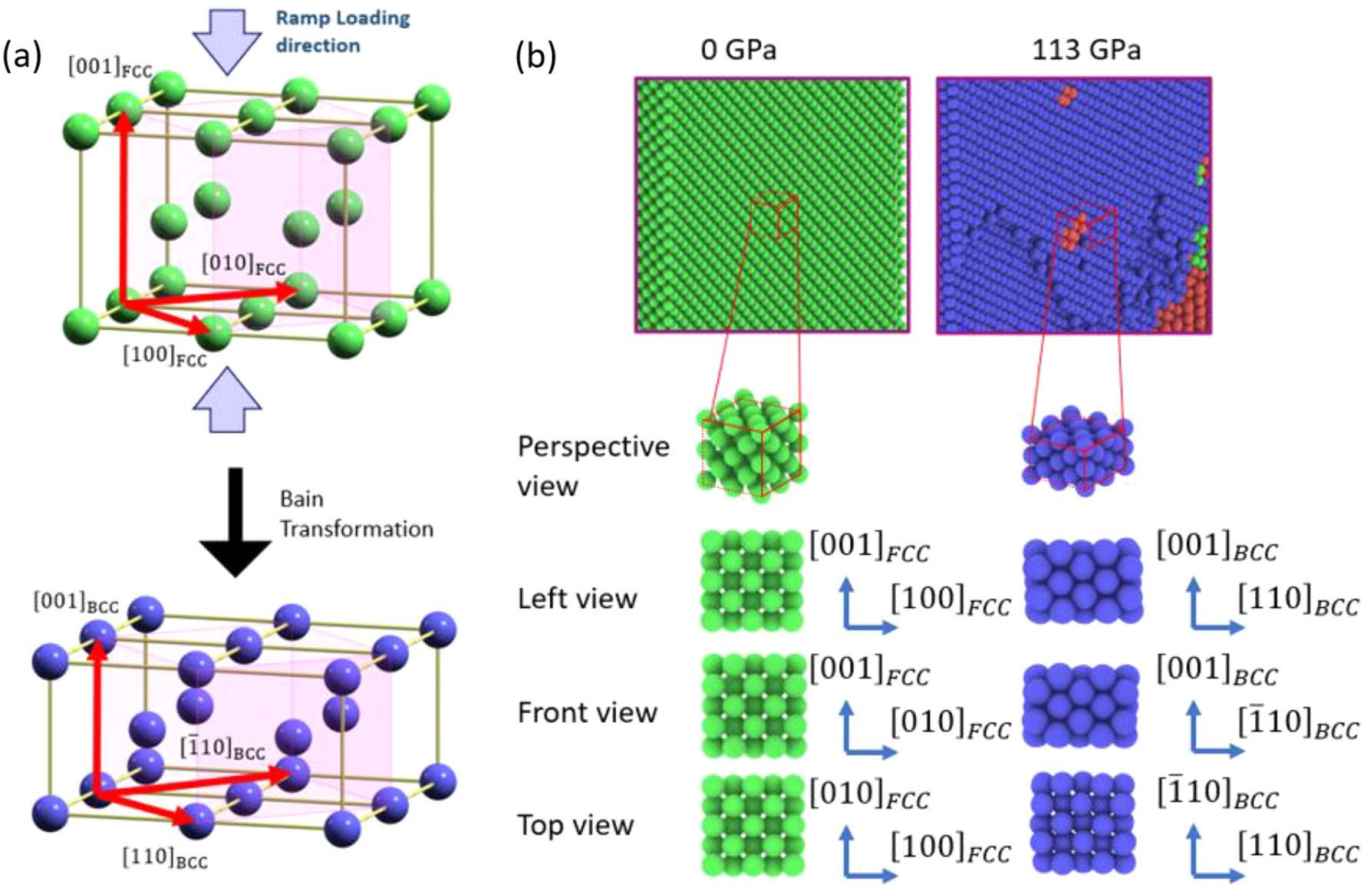
(**a**) Schematic of the Bain transformation and (**b**) Perspective, left, front and top views of a supercell that undergoes a structural phase transformation during loading at different stress and the corresponding lattice orientation.

**Figure 5:**
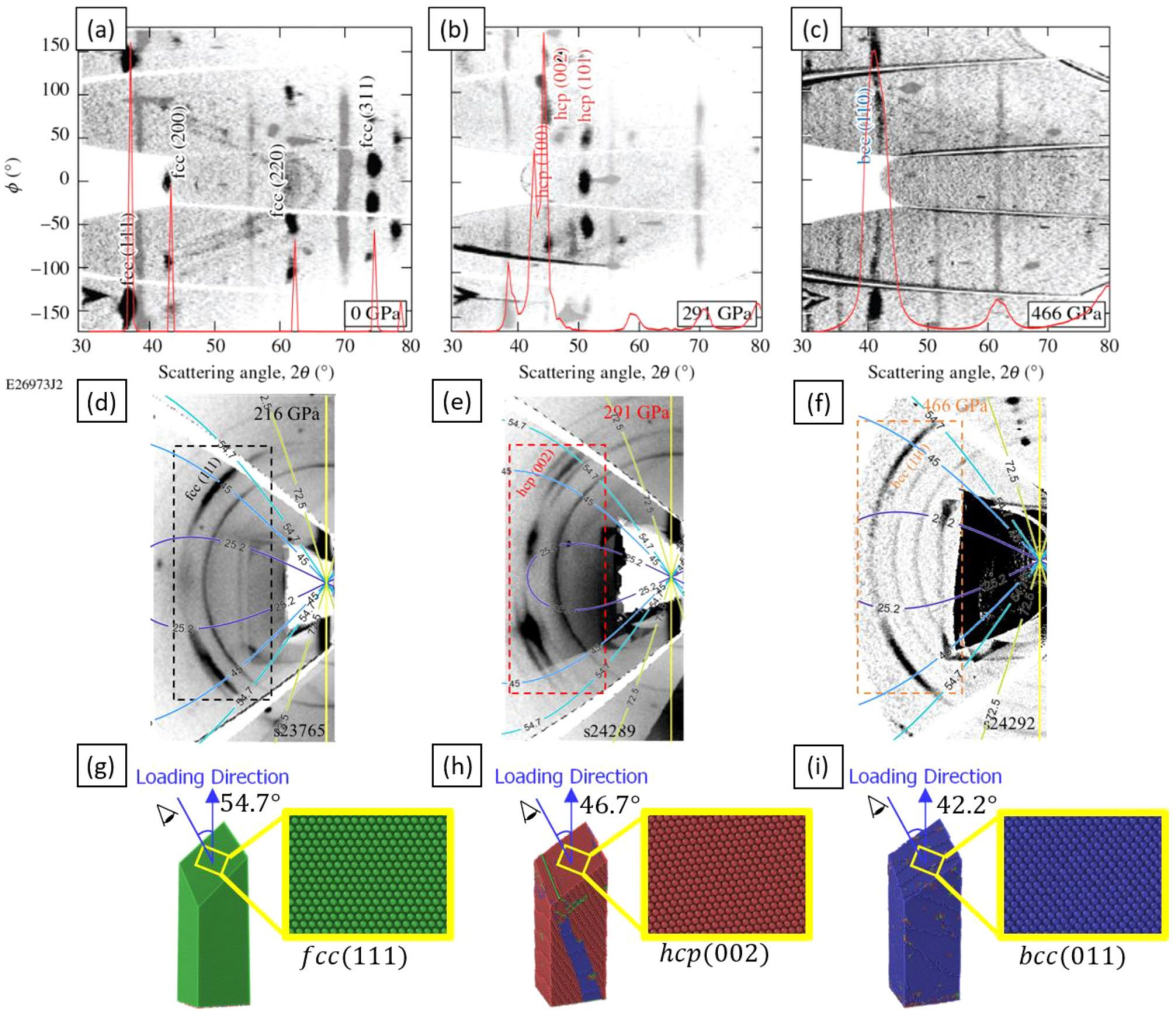
(**a**–**c**) Experiment and virtual XRD data taken at different stage of the ramp loading. The experimental data are taken at 0 GPa, 291 GPa and 466 GPa respectively. The virtual XRD data are taken at 0 GPa,76 GPa and 399 GPa respectively. Notice the single crystal diffraction Laue spots from the diamond and LiF ablator/window plates are also presented and artificially dimmed to highlight Al diffraction signature. (**d**–**f**) Stereographic projection of the diffraction data from experiment taken at different stress (216 GPa, 291 GPa and 466 GPa). The black, red, and blue boxes enclose the Debye–Scherrer rings for the fcc (111), hcp (002), and bcc (110) planes, respectively. The colored solid curves superimposed on the figures indicate constant $\chi$, (i.e., the angles between the loading direction and the diffraction plane norms). (**g**–**i**) Representative slices of the atomic configurations observed from setup II at 0 GPa, 76 GPa and 113 GPa, respectively. Coloration schme is the same as Fig. 2. The magnified areas indicate the close packed planes—fcc (111), hcp (002) and bcc (011), whose normals have angles of 54.7°, 46.7° and 42.2° with respect to the loading direction.

As stated in “Stress-density response and atomistic deformation path”, our simulations show a structural phase transformation from fcc to bcc under ramp compression. We further use polyhedral template matching^35^ method to analyze the lattice orientation of the atoms. At 0 GPa, all fcc atoms have $[100]$, $[010]$, and $[001]$ orientation along x, y, and z, respectively; at 113 GPa, all bcc atoms have $[110]$, $[\overline{1 }10],$ and $[001]$ orientation along x, y, and z, respectively. These specific lattice orientations correspond to the Bain orientation relationship (OR)^36^ between the fcc and bcc phase, as illustrated in Fig. 4a. The orientation relationship of a supercell that goes through Bain transformation is shown in Fig. 4b. The virtual XRD patterns of the 0 GPa, 76GPa, and 399 GPa are also plotted against the experimental in situ XRD took at 0 GPa, 291 GPa and 466 GPa, as shown in Fig. 5a–c. The fcc and bcc signature between experiment and simulation exhibited remarkable agreement. The lattice constant of bcc can be consequently calculated through the peak locations of Fig. 5c to be around 2.43 Å at 466 GPa for the experiment and 2.33 Å at 399 GPa for the simulation. The experiments used a polycrystalline aluminum foil that had a strong initial texture, with all grains being (001)-orientated along the fiber axis. As illustrated in Fig. 5d–f, during the deformation, the (111)_fcc_, (002)_hcp_, (011)_bcc_ spots are in close vicinity: angles $\chi$ between fiber axis (i.e., loading direction) and diffraction plane normals are ~ 45° for (111)_fcc_, ~ 50° for (002)_hcp_ and ~ 45° (011)_bcc_. Notice the theoretical angle between the (111)_fcc_ normal and the loading direction (001)_fcc_ for the ideal crystal should be 54.7°. The deviation between the theoretical value and measuremnets is due to straining and rotation of the system as a result of twinning. Thus it can be concluded from these Figures that the (111)_fcc_, (002)_hcp_, (011)_bcc_ spots are the same most close-packed planes that essentially remain parallel through the transformations. Illustrated in Fig. 5g–i are the atomic configurations observed at different stress from SC NEMD. All structures has been sliced along the close-packed plane, and the angles between the normals of these planes and the loading direction are calculated as 54.7° for (111)_fcc_, 46.7° for (002)_hcp,_ and 42.2° for (002)_hcp_, respectively, suggesting the close packed planes also remain parallel through the transformation during the simulation. For an ideal Bain transformation in strain-free structure, we would have (011)_bcc_ parallel to (111)_fcc_ and angles of 45° between the (011)_bcc_ normal and the fiber axis. To be noted, this only applies to ${\left(011\right)}_{bcc}$, ${\left(0\overline{1 }1\right)}_{bcc}$, ${\left(101\right)}_{bcc}$, ${\left(10\overline{1 }\right)}_{bcc}$, while ${\left(110\right)}_{bcc}$ and ${\left(\overline{1 }10\right)}_{bcc}$ are perpendicular to the fiber axis. These orientation analyses suggest that the phase transformation observed in the simulation matches that in the experiment. It is important to point out that the exact phase transformation can only be determined when the orientation of parallel planes as well as parallel directions inside those planes are known. Thus it is only through the NEMD simulations that we can determine the in-plane directions and the actual phase transformation path that took place during the ramp loading. Therefore, the previously proposed^15^ transformation path of Shoji–Nishiyama orientation relation^37^ for the fcc–hcp transition followed by the Burgers orientation relation^38^ for the hcp–bcc transition, which is only basd on the close pack planes and the loading axis, is not correct.

It should be noted that the transition pressure from fcc to hcp is lower in NEMD simulations in comparison to experiments. The simulated XRD profile of hcp phase is taken at 76 GPa, and in comparison, the experimental XRD is taken at 291 GPa. Although both XRD profiles for the simulation and experiment data exhibit clear hcp signatures, the relative position of peaks is shifted due to the difference in the lattice constant of the hcp phase, Fig. 5b. The difference in transition pressure between simulations and experiments could be due to advanced growth and shortened persistence of the stacking faults in the NEMD simulations, which are based on small (scaling factor 1/20 vs. 1) cells and a slightly different boundary (rigid piston vs. LiF) in comparison to samples in the actual experiments, where the effect of nucleation dynamics could extend for microns^39^. In addition, when under axial compression, the structure tends to expand along the transversal dimensions due to the Poisson’s effect. However, due to the simulation happening in a microcanonical ensemble with full periodic condictions, the transversal dimensions are not allowed to freely deform. Thus, elevated lateral stresses (at levels around 80% of the concurrent longitudinal stresses) are created on the transversal dimensions, causing the overall hydrostatic pressure in the simulation at levels comparable to the pressures measured in the experiments^15^. Therefore, one possible reason for low pressure range for hcp phase is that it consists of the stacking faults that are generated due to interaction of dislocations due to the small cross section of the sample. It has also been shown previously that hydrostatic pressure favors transformations that are in a negative volumetric change and could trigger phase transformation at lower compression stress levels^40^. All these mechanisms potentilally contribute to the lower trasition pressures observed in simulations. Further research on nucleation dynamics within solids and larger-scale NEMD simulations will be beneficial in elucidating such differences.

### Plasticity contributor in texturized nanocrystalline Al

To investigate the effect of the grain boundaries on the resultant plastic deformation behaviors, we performed atomistic ramp loading simulations on a <001> _fcc_-oriented texturized NC Al structure with similar setups to SC structures. Thus, The lateral dimensions of the texturized NC Al are three times larger than the SC to accommodate grains of the size of 15 nm.

**Figure 6:**
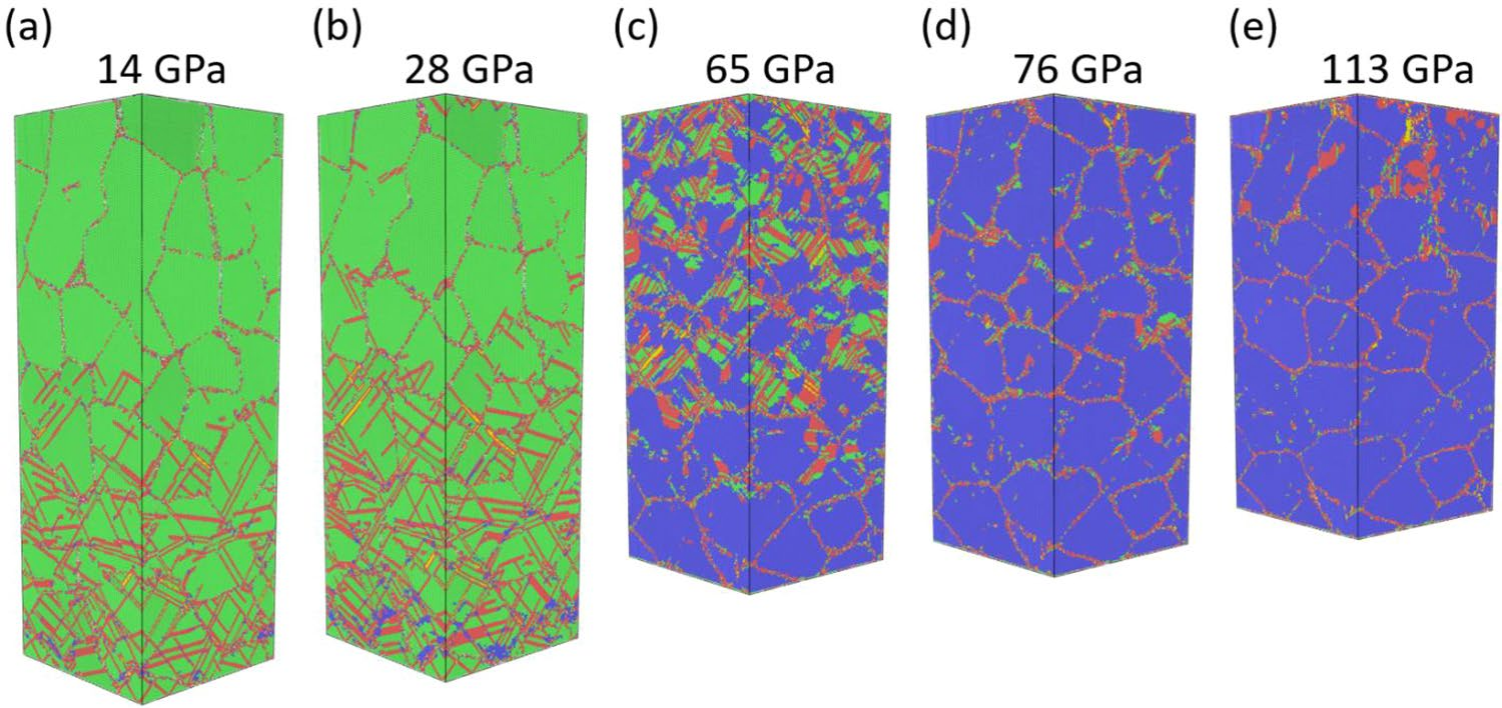
Atomistic snapshot of the texturized NC Al at different global stress. Atoms are colored in the same manner as Fig. 2. The loading direction is from bottom to top.

The stress–strain curve for the texturized NC Al is shown in Fig. 1 and exhibits a remarkable agreement to the experiments and also the SC NEMD result. Snapshots of the texturized NC Al at several critical pressures are shown in Fig. 6. It can be seen from Fig. 6a,b snapshots that the early plasticity is dominated by SFs and micro twins are almost absent as opposing to the SC strcuture. The phase transformation was also initiated and finished at smaller stresses; the entire structure transformed to bcc at 76 GPa, Fig. 6d, compared to 113 GPa for SC NEMD. Defect growth in the bcc phase is also initiated as soon as the phase transformation is completed, as illustrated by Fig. 6e. All these observations are consistent with SC NEMD, suggesting that grain boundaries have negligible effect on the ramp loading behavior. This finding is consistent with a recent study on the Hugoniot equation of state of Al^26^. Interestingly, we note that previous experiments have shown insensitivity of the Hugoniot to the grain size and orientation of copper^41^, whereas the dependence on the form of the crystal/sample is strong for diamond^42^, silicon carbide^43^, and TATB^44^. This clearly implies different mechanisms in the response of different materials to dynamic compression that are likely closely related to their metallicity.

## Conclusion

In conclusion, NEMD simulations are carried out to investigate the plastic deformation properties of Al under ramp loading conditions. Setups with varying lengths and simulation times demonstrated a scaling approach is viable when the temporal and spatial parameters are scaled identically, and the structure length is adequately long. An excellent agreement is observed in stress-density response between previously published laser-driven ramp compression experiments and simulation setups with proper scaling factors. Furthermore, the atomistic pictures and virtual diffraction analysis of a single crystal structure demonstrated a plastic deformation route via micro twin formation→SFs formation→SFs thickening→phase transformation via Bain path.

Finally, the virtual XRD patterns are compared with experimental in situ XRD results and showed remarkable similarity in the fcc and bcc signature at comparable pressures as well as similar signatures for hcp phase at different intermediate pressures. The proposed phase transformation path is also cross-examined with the experimental diffraction result and showed good agreement. This study provided concrete evidence of the exact phase transformation path for Al that took place during ramp loading and also provided insights into understanding experimental diffraction results by correlating the analysis of the virtual diffraction patterns with atomistic configurations.

## Methods

The NEMD simulations are carried out using the Large-scale Atomic/Molecular Massively Parallel Simulator (LAMMPS)^45^ code. Before the loading, all structures underwent energy minimization using the conjugate gradient method with a maximum force tolerance of 10^−27^ eV/Å. Then two relaxation runs are carried out, first under zero pressure and room temperature in a Nosé–Hoover isothermal–isobaric (NPT) ensemble and then in a microcanonical (constrained energy and volume NVE) ensemble. OVITO^46^ is employed for post-processing of the simulation results and for the visualization of atomistic snapshots; Polyhedral Template Matching^35^ is used for crystal structure and orientation identification; Dislocation Extraction Algorithm (DXA)^47^ tool is used for dislocation analysis; Virtual XRD implemented in LAMMPS are employed to generate diffraction signature of the atomistic snapshot at any given time. The virtual XRD irradiation wavelength is set at either 1.48 Å or 1.21 Å to compare with the experiments (8.37-keV (Cu) and 10.25-keV (Ge) He- α) directly.

## Supplementary Information

Supplementary Information.

## Data Availability

The datasets used and/or analysed during the current study available from the corresponding author on reasonable request. Sample of lammps scripts and related potential files are provided in the following link: https://rochester.box.com/s/loo1ks1jn98mej6khzecq7w7jr2lapcp.
