# Supplementary material for: Phase transformation path in Aluminum under ramp compression; simulation and experimental study
Source: Sci Rep. 2022 Nov 8;12:18954. doi: 10.1038/s41598-022-23785-7 (PMC9643319; doi:10.1038/s41598-022-23785-7)
Supplement: Supplementary file 1 — Supplementary Information. [file 41598_2022_23785_MOESM1_ESM.docx]

Phase transformation path in Aluminum under ramp compression; simulation and experimental study - supplementary materials

Lijie He^1^, Danae Polsin^2,3^, Shuai Zhang^3^, Gilbert W. Collins^2,3,4^, Niaz Abdolrahim^1,2^

Materials Science Program, University of Rochester, Rochester, NY 14627, USA

Departments of Mechanical Engineering, University of Rochester, Rochester, NY 14627, USA

Laboratory for Laser Energetics, University of Rochester, Rochester, NY 14623, USA

Department of Physics and Astronomy, University of Rochester, Rochester, NY 14627, USA

1. Dynamic scaling of the ramp loading system^1, 2^

Assuming one-dimensional flow in a ramp loading system, the piston trajectory can be scaled linearly in both coordination and time by a factor of $1/M$:

$x_{s}\left( t_{s} \right)=\frac{1}{M}x\left( t \right)$ (1)

$t_{s}=\frac{1}{M}t$ (2)

where *x* denotes coordination, *t* denotes time; variables with subscript *s* indicate scaled values. Doing such will ensure the piston velocity under the scaled coordination remains the same:

$v_{s}\left( t_{s} \right)=\frac{\partial x_{s}}{\partial t_{s}}=\frac{\frac{1}{M}\partial x}{\frac{1}{M}\partial t}=\frac{\partial x}{\partial t}=v(t)$ (3)

The ratio between the forces of the scaled system and the original system satisfies:

$\frac{F_{s}}{F}=\frac{m_{s}(L_{s}/T_{s}^{2})}{m(L/T^{2})}=\frac{\rho_{s}A_{s}L_{s}(L_{s}/T_{s}^{2})}{\rho AL(L/T^{2})}=\left( \frac{\rho_{s}}{\rho} \right)\left( \frac{L_{s}}{L} \right)^{2}\left( \frac{T_{s}}{T} \right)^{-2}$ (4)

where $m$, $L$, $T$, $\rho$, $A$ denotes mass, length, time, density, and cross-section area. Since the scaling only happens on one dimension,

$A_{s}=A$ (5)

The ratio between scaled and original length, time, and density can be acquired through equation (1):

$\frac{L_{s}}{L}=\frac{T_{s}}{T}=\frac{1}{M}$ (6)

$\frac{\rho_{s}}{\rho}=1$ (7)

plugging into equation (4),

$\frac{F_{s}}{F}=1$ (8)

which suggests the forces are invariant to the proposed scaling. As a result, no modification towards the interatomic potential is needed. Similarly, velocity, strain, stress, density, and temperature are all invariant to the proposed scaling. On the contrary, acceleration, strain rate, and any extensive variable are not invariant. The scaling method is only strictly valid when the spatial and temporal scaling parameters are equal. A dimensionless strain rate ${\dot{\tilde{v}}}_{p}$ is proposed by Lane et al.^2^, to identify systems that satisfy this prerequisite:

${\dot{\tilde{v}}}_{p}=\frac{v_{t}L}{\tau C_{0}^{2}}$ (9)

where $v_{t}=6 km/s$ is the terminal velocity for both the experiment and the simulation. $\tau$ denotes the acceleration duration,$L$ is the piston length and $C_{0}=6.27 km/s$ is the ambient sound velocity of Aluminum. According to the scaling method, setups with the same dimensionless strain rate have identical temporal and spatial scaling factors and thus satisfy the scaling prerequisite. It is worth pointing out that the scaling method also has certain limitations: first, it only works well under quasi-one-dimensional simulation setups. Changes in the lateral dimensions could potentially annihilate the invariance of force and introduce significant errors. Second, it is proven by Thompson et al.^1^ that the scaling method does not strictly apply to the plastic regime, especially when the overall system size is too small to produce reliable statistics. Nevertheless, they reported excellent scaling in the plastic regime, suggesting that the scaling method could be viable, but system size and plastic deformation must be carefully considered.

To verify the scaling method, we have conducted multiple ramp-loading molecular simulations on different <001>-oriented single-crystal (SC) and texturized nanocrystalline (NC) with different scaling factors, as summarized in table 1. For the single crystal setup, an initial <001>-oriented SC Al system with varying sizes is created and ramp-compressed in the Z direction; pistons are set initially at the lower Z boundary and move up with linearly increasing velocity up to 6 km/s. However, the acceleration duration is different for each setup. The lateral size (along the transverse directions x and y) in SC models is set at $10.12 nm,$ while the length (along the loading direction z) varies from $500 nm$ to $2 microns$. All other settings are the same as in the main text. The deformation behaviors of different setups are discussed in detail in section 3 of this supplementary material. An additional SC structure with the same setting as setup II except for three times larger lateral size ($30.37 nm$) has also been tested and compared with setup II. No difference was observed in their macroscopic mechanical response and microscopic deformation mechanisms, indicating that the structure’s lateral dimension is large enough to produce reliable and consistent results.

Table 1 Experiment and simulation setups. Within the duration of each, the piston velocity linearly ramps from zero up to 6 km/s.

| **Setup** | **Length(nm)** | **Duration(ps)** | **Length Scale Factor** | **Time Scale Factor** | **Atom**  **Count** | **Dimensionless strain rate** |
| --- | --- | --- | --- | --- | --- | --- |
| Polsin (Experiment) | 20000 | 10000 | 1 | 1 | N/A | 0.306 |
| I | 100 | 50 | 1/200 | 1/200 | 6.25×10^5^ | 0.306 |
| II | 1000 | 500 | 1/20 | 1/20 | 6.25×10^6^ | 0.306 |
| III | 2000 | 1000 | 1/10 | 1/10 | 1.25×10^7^ | 0.306 |
| IV | 1000 | 250 | 1/20 | 1/40 | 6.25×10^6^ | 0.612 |
| V | 2000 | 250 | 1/10 | 1/40 | 1.25×10^7^ | 1.224 |
| VI | 500 | 500 | 1/40 | 1/20 | 6.25×10^6^ | 0.153 |
| VII | 2000 | 500 | 1/10 | 1/20 | 1.25×10^7^ | 0.612 |
| Texturized NC | 100 | 50 | 1/200 | 1/200 | 5.63×10^6*^ | 0.306 |

* The texturized NC structure possess a larger lateral size, thus having disproportional large atom count.

1.
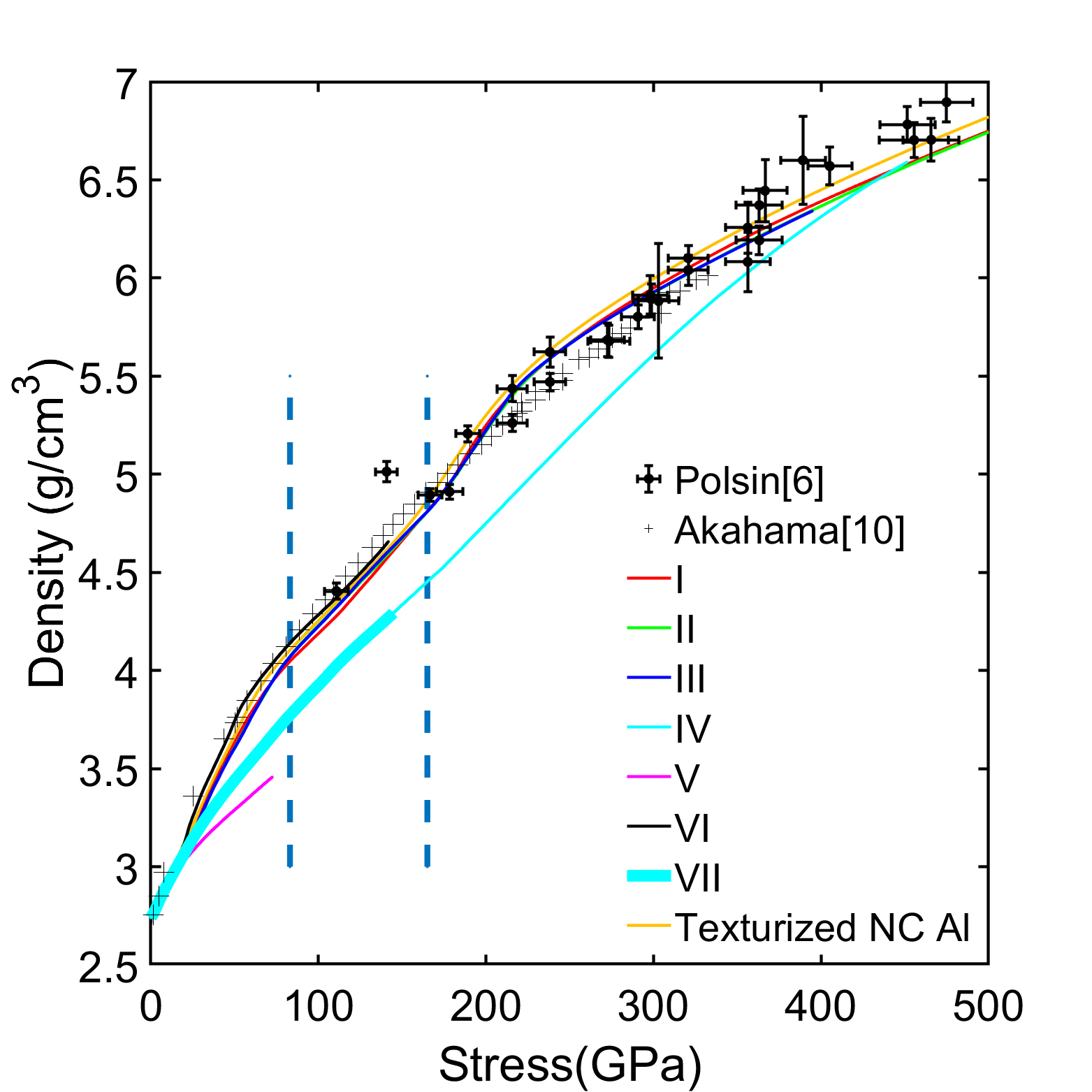
Stress-density response with different scaling factors.

Figure S1 Density–stress curves for different simulation setups. The data are compared with ramp experiment from Ref [6] and diamond anvil cell data from Ref [10] of the main text. For both the simulation data and ramp experiment data, the stress and density refers to the average stress and density throughout the thickness of thes entire Al domain as a function of time, which will be referred to as the global stress and density for the rest of the paper. The first vertical dashed bar denotes the pressure onset for 100% bcc in setups II and III. In between the two vertical dashed bars, the structure is full bcc and deforming elastically for setup II and III. After the second dashed bars, defect growth is observed in setup II and III. For setup I, 100% bcc is triggered at a lower stress, causing the curve to deviate from setups II and III in between the two vertical bars.

In this section, the stress-density response and deformation behaviors of all SC setups are discussed and compared in detail. As illustrated in Table 1, setups I, II and III have the same dimensionless strain rate as the experimental setup (i.e., the length and time scale factor are equal for each setup). In contrast, setup IV, V and VII possess larger dimensionless strain rates while setup VI possess smaller dimensionless strain rate. As shown in Figure 1, the stress-density curves of setups I, II, III and VI are in very good agreement with both the ramp loading experiment and diamond anvil cell data, while setups IV, V and VII are deviated from the experimental results due to having the dimensionless strain rate being too large. Closer inspection reveal that in setup I, II, III and VI, the elastic wave have time to develop and propagate in the structure while in setup IV, V and VII, the plastic wave develop much quicker, comparable with the elastic wave, and the atoms have less time to relax, causing the higher stress observed.

For setup I with a smaller dimensionless strain rate, although the stress-density curve is very similar to setup II and III, the phase transformation is triggered and complete at smaller stresses. the entire structure transformed to bcc at 76 GPa, compared to 113 GPa for the larger scale factors (setup II and III). Furthermore, the new bcc phase in setup I exhibits a non-negligible amount of point defects (a minimum of 3.6% of the atoms at 102 GPa and keep increasing); in comparison, the bcc phase in setups II and III was almost defect-free (<0.1% atoms) from 100 GPa to 165 GPa. This difference in the bcc phase behavior leads to a subtle deviation in the density-pressure curves between the aforementioned stress range, as illustrated in Figure S1. Thus, it is concluded that changing the scaling method could affect the observed plastic deformation mechanims. Thompson et al.^1^ have also observed similar behavior and concluded that finite system size effects in plastic regimes could invalidate the scaling method and conclude that making the system larger could mitigate this issue. Setup II (scaling factor 1/20) behaves identically as setup III (scaling factor 1/10) during the entire loading process, indicating a scaling factor of 1/20 is sufficient for the prediction of proper plastic deformation behavior using the scaling method. As a balance between model accuracy and computational efficiency, setup II is adopted as the focus of this investigation and presented as the SC NEMD data in the main text.

1. Mattsson, T. K. R.; Desjarlais, M. P.; Grest, G. S.; Templeton, J. A.; Thompson, A. P.; Jones, R. E.; Zimmerman, J. A.; Baskes, M. I.; Winey, J. M.; Gupta, Y. M.; Lane, J. M. D.; Ditmire, T.; Quevedo, H. J. *Modeling ramp compression experiments using large-scale molecular dynamics simulation*; United States, 2011-10-01, 2011.

2. Lane, J. M. D.; Foiles, S. M.; Lim, H.; Brown, J. L., Strain-rate dependence of ramp-wave evolution and strength in tantalum. *Physical Review B* **2016,** *94* (6), 064301.
